# The impact of HIV-1 on the malaria parasite biomass in adults in sub-Saharan Africa contributes to the emergence of antimalarial drug resistance

**DOI:** 10.1186/1475-2875-7-134

**Published:** 2008-07-22

**Authors:** Jean-Pierre Van geertruyden, Joris Menten, Robert Colebunders, Eline Korenromp, Umberto D'Alessandro

**Affiliations:** 1 Department of Parasitology, Unit of Epidemiology Institute of Tropical Medicine Antwerpen, Nationalestraat 155 B2000, Antwerpen, Belgium; 2 Department of Public Health, Institute of Tropical Medicine, Nationalestraat 155, B-2000, Antwerp, Belgium; 3 Department of Clinical sciences, Institute of Tropical Medicine and University of Antwerp, Antwerp, Belgium; 4 Department of Public Health, Erasmus MC, University Medical Centre Rotterdam, Rotterdam, The Netherlands; 5 Performance Evaluation and Policy unit, The Global Fund to Fight AIDS, Tuberculosis and Malaria, Geneva, Switzerland

## Abstract

**Background:**

HIV-related immune-suppression increases the risk of malaria (infection, disease and treatment failure) and probably the circulating parasite biomass, favoring the emergence of drug resistance parasites.

**Methods:**

The additional malaria parasite biomass related to HIV-1 co-infection in sub-Saharan Africa was estimated by a mathematical model. Parasite biomass was computed as the incidence rate of clinical malaria episodes multiplied by the number of parasites circulating in the peripheral blood of patients at the time symptoms appear. A mathematical model estimated the influence of HIV-1 infection on parasite density in clinical malaria by country and by age group, malaria transmission intensity and urban/rural area. In a multivariate sensitivity analysis, 95% confidence intervals (CIs) were calculated using the Monte Carlo simulation.

**Results:**

The model shows that in 2005 HIV-1 increased the overall malaria parasite biomass by 18.0% (95%CI: 11.6–26.9). The largest relative increase (134.9–243.9%) was found in southern Africa where HIV-1 prevalence is the highest and malaria transmission unstable. The largest absolute increase was found in Zambia, Malawi, the Central African Republic and Mozambique, where both malaria and HIV are highly endemic. A univariate sensitivity analysis shows that estimates are sensitive to the magnitude of the impact of HIV-1 infection on the malaria incidence rates and associated parasite densities.

**Conclusion:**

The HIV-1 epidemic by increasing the malaria parasite biomass in sub-Saharan Africa may also increase the emergence of antimalarial drug resistance, potentially affecting the health of the whole population in countries endemic for both HIV-1 and malaria.

## Background

Since the 1980s, the epidemiological overlap of HIV-1/AIDS and malaria in tropical regions, and particularly in Eastern and Southern Africa, has been cause for concern as even a small interaction between the two diseases may be of great public health importance [1-4]. HIV-1 and malaria coexist in regions where the health surveillance systems are poorly performing so that the magnitude of any interaction is difficult to determine. Nevertheless, the evidence of such interaction has recently grown and is still increasing. The incidence of symptomatic malaria episodes, severe or uncomplicated, and the corresponding parasite density is higher in HIV-1 infected individuals with low CD4 count [5].

It has been recently stated that, in Sub-Saharan Africa, the direct impact of HIV-1 infection on malaria morbidity and mortality is limited as the two diseases do not have the same geographical distribution and affect different age groups [6]. However, a model assessing the impact of HIV-1 on malaria in sub-Saharan Africa, estimated that in areas of unstable malaria transmission, such as southern Africa, the HIV-1 epidemic contributed to the increase of malaria observed in the 1990s. Moreover, a recent mathematical model indicated a significant role for the dual infection in fueling the spread of both diseases in sub-Saharan Africa [7]. Besides a direct effect, HIV-1 infection may indirectly influence the malaria burden by increasing the malaria parasite biomass and consequently the probability of drug resistant parasites emerging. Antimalarial drug resistance, particularly for *Plasmodium falciparum*, is considered a major contributor to the global resurgence of malaria observed over the last three decades [8] and one of the greatest obstacles for an effective malaria control [9]. The basis of resistance lies in one or several genetic mutations in the parasite genome. Malaria parasites with such mutations when in contact with a given drug survive the treatment and eventually spread. Assuming that the occurrence of a *de novo *resistance mutation is evenly distributed among all parasites, a larger parasite biomass would increase the probability of new resistant strains emerging. Therefore, the additional malaria parasite biomass related to HIV-1 infection was estimated by reviewing the available literature on the impact of HIV-1 infection on malaria morbidity (incidence of clinical cases and parasite density) at individual level, and HIV-1 prevalence and malaria transmission intensity data at country level. The results of this analysis and the possible consequences of HIV-1 on the emergence of antimalarial drug resistance are reported and discussed below.

## Methods

### Definition of endemicity

The MARA ("Mapping Malaria Risk in Africa") climate suitability index was used for determining the country endemicity, i.e. high ≥ 0.75 and low <0.75 [10]. However, Botswana, Namibia, South Africa, Swaziland, and Zimbabwe, because of their successful vector control programme, were not classified according to this criterion and despite a climate suitability index >0.75 were considered as low rather than high transmission areas [10].

### Malaria incidence and parasite density without HIV-1 infection

Incidence rates of symptomatic malaria (uncomplicated and severe) by transmission intensity and age group were collected from the available literature [6]. In high endemic rural areas the average incidence was estimated at 1.4 malaria episodes/person-year (py) among children <5 years of age, 0.59/py in the 5–14 year old and 0.11/py in adults (≥ 15 year old)[10]. In low endemic rural areas, incidence was estimated at 0.18/py in children <15 years old and 0.09/py in those aged 15 years and above (Table 1). In high endemic urban areas, the malaria incidence was estimated to be similar to that in low endemic rural areas while in low endemic urban areas, the malaria incidence was estimated at half of the low endemic rural areas [11] (Table 1). In Botswana, Namibia, South Africa, Swaziland, and Zimbabwe, the average incidence was estimated at 0.03/py [10], with the age pattern distribution similar to that of low endemic areas [6]. The proportion of urban dwellers was assumed not to vary by malaria endemicity. Urban and rural populations were based on countries' definitions, with no standardization between countries [12].

**Table 1:** Assumptions on malaria incidence rates, parasite density, and effects of HIV-1, sub-Saharan Africa*

	**Age group**		
***HIV-negative persons and HIV-infected persons with CD4 ≥ 500/μl***	**<*5 years***	***5–14 years***	***≥ 15 years***

Incidence of symptomatic malaria (per person-year) low/high endemicity[10]			
Rural	0.18/1.4	0.18/0.59	0.09/0.11
Urban	0.09/0.18	0.091/0.18	0.046/0.09
Proportion of symptomatic episodes with hyperparasitaemia (includes severe malaria): low endemic and high endemic urban areas/high endemic rural areas	4.0%/4.0%	4.0%/1.5%	4.0%/1.5%
Geometric mean parasite density (/μL) in uncomplicated malaria: low endemic and high endemic urban areas/high endemic rural areas [13]	30,000/30,000	25,000/20,000	20,000/15,000
Geometric mean parasite density (/μL) in severe malaria (all age groups and endemicities) [20]	458 000		
Proportion of HIV-1 patients [6]	*with CD4<200/μl*	CD4 200–499/μL	
Stabilized HIV epidemic	37%	25%	
Rising HIV epidemic (e.g.Madagascar)	22%	15%	
***Relative change in HIV infection***	***with CD4<200/μl***	**CD4 200–499/μL**	
Relative risk of symptomatic malaria incidence (all age groups and endemicities) [1,21-24]	5.0	3.0	
Relative parasite density in symptomatic malaria (all age groups and endemicities) (Table 2)	3.0	1.5	
Relative risk of progression to severe malaria (all age groups and endemicities) [16,25,26]	15.0	6	
Relative parasite density during severe malaria (all age groups and endemicities)	1.0	1.0	

Geometric mean parasite density in falciparum malaria patients was usually between 5,000 and 50,000/μl, with the lowest densities found where transmission was low and the highest where transmission was high [13]. In low endemic and in high endemic urban areas, the mean parasite density in clinical cases was assumed to be 30,000/μl in children <5 years old, 25,000/μl in those aged 5–14 years and 20,000/μl in patients aged ≥ 15 years. In high endemic rural areas, the mean parasite density in clinical cases was assumed to be 30,000/μl for children <5 years old,, 20,000/μl for those aged 5–14 years, and 15,000/μl for patients aged ≥ 15 years (Table 1). To allow the calculation of the population-level parasite biomass, the geometric mean parasite densities were converted into arithmetic means, by assuming that parasite densities in symptomatic malaria have an exponential distribution [14]. Arithmetic mean densities were therefore estimated at 51,347/μl for children <5 years old, 42,789/μl for those aged 5–14 years, and 34,231/μl for patients aged ≥ 15 years in low endemic and high endemic urban areas; in high endemic rural areas the corresponding figures by age group were 51,347/μl, 34,231/μl and 25,678/μl.

A proportion of symptomatic malaria episodes evolves towards severe disease [15], generally with higher parasite densities than uncomplicated episodes. However, the sequestration of *P. falciparum*-infected erythrocytes in the deep circulation confounds the relationship between peripheral parasite density and disease severity [16-19]. It was assumed that 4% of clinical episodes both in low transmission (all age groups) and high (children) transmission areas evolve towards high parasite densities (some of them would have severe malaria). In high-transmission areas, 1.5% of all adult malaria episodes were assumed to be hyperparasitaemic at enrolment. Based on a recent model[20], these patients were assumed to have a geometric mean parasite density of 458,000 parasites/μL (309,659–678,910), corresponding to an arithmetic mean of 784,000 parasites/μL (530,000–1,162,000). Average blood volumes were estimated for each age group on the basis of heights and weights, i.e. one liter in the <5 years old, two liters for the 5–14 years old and five liters for the ≥ 15 years old (range across human populations 0.25–8 liters).

### Malaria incidence and parasite density with HIV-1 infection

The best evidence for an association between HIV infection and incidence of symptomatic malaria comes from four longitudinal population-based studies in rural Uganda and Malawi, where malaria is highly endemic. In Uganda, odds ratios of symptomatic malaria in HIV-infected compared to uninfected adults were 6.0, 3.4 and 1.2 for CD4 counts <200/μl, 200–499/μl and ≥ 500/μl, respectively [1]. In another study in Uganda, the incidence of symptomatic malaria in HIV-infected individuals varied according to CD4 count and was 0.14/py, 0.093/py and 0.057/py for CD4 count of <200/μL, 200–499/μL and >500/μL, respectively (0.09/py, 0.053/py, and 0.022/py when considering only episodes with more than 2,800 parasites/μL)[21]. In Malawi, incidence rates of symptomatic malaria in HIV-1 infected adults varied with CD4 count; compared to individuals with CD4 count of ≥ 500, the malaria incidence was 3-fold higher with a CD4 count of 200–499/μL and a 4.4-fold higher with a CD4 count <200/μL [22]. Another study from Malawi reports malaria incidence of 3.2/py, 4.9/py and 5.4/py in HIV-1 infected adults with CD4 count of ≥ 400/μL, 200–399/μL and <200/μL, respectively [23]. In most of these studies malaria infection was not assessed at the time that CD4 count was done. This is a limitation as malaria itself causes a temporarily and reversible decrease in peripheral CD4 count (by one third) [24]. Therefore, the risk for clinical malaria may increase at a higher-than-expected CD4 count. We assumed that, compared to HIV-1 negative individuals, the risk of clinical malaria in HIV-1 infected individuals with a CD4 count <200/μl and 200–499/μL is 5 and 3-fold higher, regardless of endemicity and age. HIV-1 infected with a CD4 count ≥ 500 were expected to have no additional risk.

HIV-1 infection considerably increases the risk of severe malaria in adults [16,25,26]. In South Africa, in an area of low (unstable) transmission, malaria-attributable mortality was nearly 7-fold higher in HIV-infected malaria patients. Similar observations have been collected in an ongoing study in a meso-endemic region of Zambia, with the highest risk in HIV-1 infected with low CD4 count (Chalwe, unpublished data). We assumed that, compared to HIV-1 negative individuals, the risk of severe malaria in HIV-1 infected individuals with a CD4 count <200/μl and 200–499/μL is 15 and 6-fold higher, regardless of endemicity and age. HIV-1 infected with a CD4 count ≥ 500 were expected to have no additional risk.

### Parasite density in clinical malaria and HIV-1 infection

HIV-1 infection tends to increase the mean parasite density, although this effect is largely limited to patients with advanced immune suppression (Table 2). In HIV-1 infected individuals, the geometric mean parasite density in uncomplicated malaria was assumed to be three-fold higher at CD4 count <200/μl and 50% higher at CD4 count 200–499/μL, irrespective of malaria endemicity and patient's age. For severe malaria, because parasite density is already high and few data are available, it was conservatively assumed that parasite density does not vary according to HIV infection.

**Table 2:** Overview of African studies on geometric mean parasite densities during uncomplicated symptomatic malaria, by HIV-1 status of the patient

**Study**	**Setting**	**Median** **age** ** (range)**	**# patients (or ** **malaria episodes,** ** if indicated)**	**Parasite density, ** **HIV-positive *vs*. HIV negative patients**			**Parasite density in HIV-infected:** immune-suppressed *vs*. not immune-suppressed*		
**CHILDREN**	**HIGH ENDEMICITY**			**Values**	**% change**	**P-value**	**Values**	**% change**	**P-value**

Shaffer [45]	Zaire, 1988	3.3 y	166 (6 HIV+)	93 325 *vs *8 913/μL	947%	*0.04*	---		
Mermin, 2004 [46]	Uganda, 2001–3	0–5 y	NR	154 *vs *61/200 WBC	152%	*0.03*	---		
Kamya, 2006 [47]	Uganda, 2002–4	3 y	1802	28 186 *vs *20 076/μL	40%	*NS*	---		
Otieno, 2006 [19]	Kenya, 2003–4	11 m	317	48 356 *vs *47 245/μL	2%	*NS*	---		
Greenberg, 2001 [17]	DRC, 1986–8	5–21 m	271 episodes	4 173 *vs *3 854/μL	8%	*NS*	AIDS *vs*. HIV positive: 11256 vs 2394/μL	370%	*0.068*
Taha, 1994 [48]	Malawi, 1989	0–18 m	564 episodes	---		*NS*	---		
Colebunders, 1990 [49]	Zaire, 1986–7	7 y	59	3 210 *vs *4 170/μL	-23%	*NS*	---		
Muller, 1990 [50]	Uganda, 1989	11 m	75	---		*NS*	---		

**ADULTS**	**HIGH ENDEMICITY**								

Mermin, 2004 [46]	Uganda, 2001–3	34 y	90 episodes	---		*NS*	<200 vs. ≥ 200 CD4/μL: 60 vs. 24/200 WBC	185%	*0.02*
French [21]	Uganda, 1995–98	31 y	153 episodes	---			Association low CD4 ↔ high parasite density		*0.0001*
Kamya, 2006 [47]	Uganda, 2002–4	28 y	163	20 537 *vs *15 174/μL	35%	*NS*	---		
Laufer, 2006 [22]	Malawi, 2002–3	31.7 y	203	---			Association low CD4 ↔ high parasite density		*0.005*
Van geertruyden, 2006 [37]	Zambia, 2003–5	27 y (15–50)	971	8 715 *vs *8 538/μL	2%	*NS*	<300 vs. ≥ 300 CD4/μL: 10 093 vs. 7328/μL	37%	*0.03*
Shah, 2006 [38]	Kenya, 2002–4	Adults	619	11 732 *vs *9 516/μL	22%	*NS*	<200 vs ≥ 200CD4/μL: 22 515 vs. 8232/μL	173%,	*<0.005*
Francesconi, 2001 [18]	Uganda, 2000	25 y	170	---		*NS*	---		
Whitworth, 2000 [1]	Uganda, 1990–98	35 y	559 episodes	---		*NS*	Association low CD4 ↔ high parasite density		*0.02*
Simooya [51]	Zambia, 1988	>12 y	170	---		*0.10*	---		
Muller, 1990 [50]	Uganda, 1989	26 y	200	---		*NS*	---		

**CHILDREN**	**LOW ENDEMICITY**								

Kalyesubula, 1997 [18]	Uganda, 1988	0–4 y	635 episodes	---		*NS*	---		
Grimwade, 2003 [52]	South Africa, 2000	7 y	663	---		*NS*	---		

**ADULTS**	**LOW ENDEMICITY**								

Atzori, 1993 [2]	Tanzania, 1991	18–41 y	300	---		*NS*	---		
Grimwade, 2004 [16]	South Africa, 2000	30 y	613	16.7% *vs *12.5%*	34%	*036*	---		
Birku, 2002 [36]	Ethiopia, 2000	31 y	19	27 486 *vs *32 892/μL	-16%	*NS*	---		

Abbreviations: NS = non-significant; --- = Not reported; m = months; y = years; RR = relative risk; WBC = white blood cells. *All studies except for [23] measured CD4 count during malaria episodes, which, due to the temporary decrease in CD4 caused by symptomatic malaria [24], probably inflated the CD4 threshold below which HIV-related immune-suppression starts to increase malaria incidence.

### HIV-1 prevalence and CD4 counts in HIV-infected individuals

Point estimates of national HIV-1 prevalence for 2005 are available from the Joint United Nations Programme on HIV/AIDS (UNAIDS) [27]. The impact of HIV infection was evaluated separately for urban and rural areas, because of varying intensity of malaria transmission. The urban-to-rural ratios in HIV-1 prevalence were estimated from national household surveys or antenatal clinic surveillance data [27,28]. For all countries, except Madagascar, it was assumed that 37% and 25% of HIV-infected people have a CD4 count <200/μL and 200–499/μL respectively, a good approximation for the African populations where HIV prevalence has stabilized [6]. For Madagascar, where adult HIV prevalence is low but rising, 37% of HIV-infected people were assumed to have a CD4 count <500 μL [6].

### HIV-1 infection and parasite biomass increase at population level

Annual parasite biomass was defined as the incidence rate (per person-year) of symptomatic (uncomplicated and severe) episodes multiplied by the total number of parasites circulating in the patient's peripheral blood at the time of the clinical episode (i.e. when parasite density is measured at a health facility). The parasite biomass was estimated for each country by age group (<5, 5–15 and above 15 years), by urban/rural and by low and high endemicity taking into account the HIV-1 prevalence, the incidence rates of uncomplicated and severe (hyperparasitaemic) malaria and their corresponding parasite density. The overall country parasite biomass was computed by summing up the estimations for each category listed above.

To assess the sensitivity of the results, both univariate and multivariate sensitivity analyses were performed. In the univariate sensitivity analysis, the impact of HIV was recalculated for two alternative scenarios, corresponding to the lower and upper bound of the likely range. In the multivariate sensitivity analysis, 95% confidence intervals were calculated using the Monte Carlo simulation in which 1,000 samples were taken from triangular distributions for all parameters [29] and the resulting proportional increase in parasite was calculated. All statistical analyses were performed using Microsoft Excel and the statistical software R version 2.2.1 [30].

## Results

**Figure 1:**
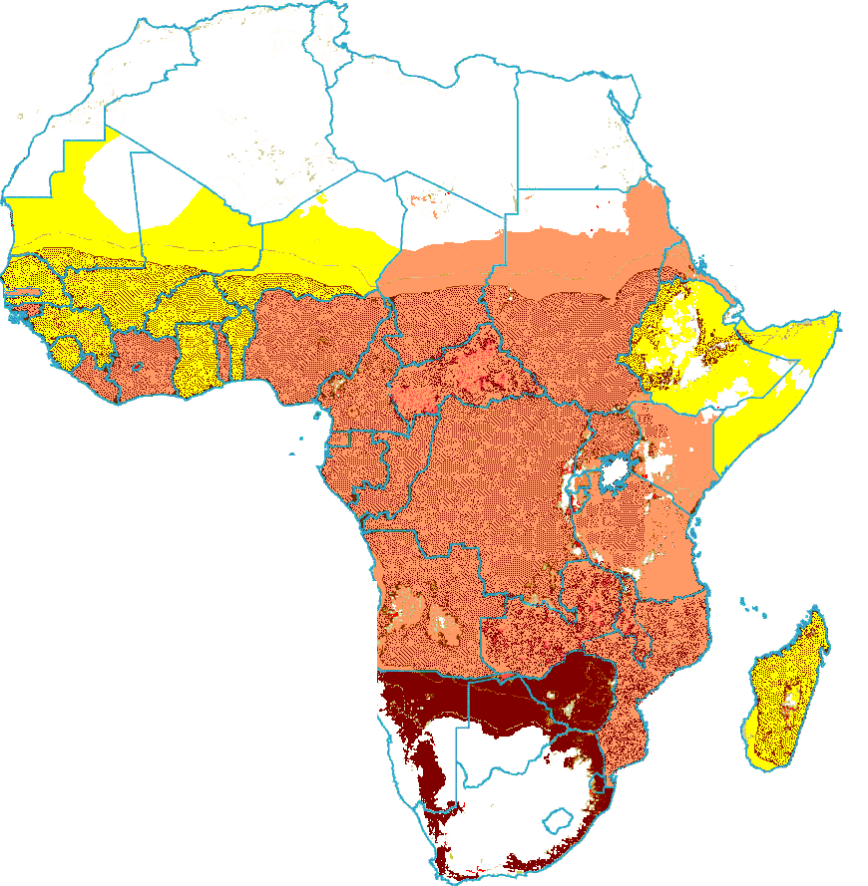
**Additional parasite biomass due to HIV-1 in individuals living in malaria risk**. No Malaria Transmission . < 10% Parasite Biomass increase* . 10–99% Parasite Biomass increase* . ≥ 100% Parasite Biomass increase* . *High Malaria Transmission areas shaded.

In sub-Saharan Africa (41 countries), the HIV-1 epidemic increases the malaria parasite biomass on average by 18.0% (95%CI: 11.6–26.9), ranging from 2.0% in Madagascar to 243.9% in Swaziland. The highest proportional increases were found in Southern African countries, where HIV prevalence is the highest and malaria transmission unstable (Table 3, Figure 1).

**Table 3:** Estimated impact of HIV on malaria parasite biomass, sub-Saharan Africa, 2005*

**Country**	Adult HIV prevalence	Urban^†^	% low-high malaria endemic	Parasite biomass*10^9^ /individual at risk in the absence of HIV^‡^	HIV-related additional parasite biomass*10^9^ /individual at risk^‡^	Proportional increase in parasite load due to HIV (95%CI)
**Mozambique**	16.1%	38%	4 – 96	77.2	54.3	64.0 (41.0–95.8)
**Zambia**	17%	36.5%	16 – 83	67.8	42.8	59.1 (35.3–94.3)
**Malawi**	14.1%	17.2%	22 – 77	75.3	40.2	54.1 (26.4–96.8)
**CARepublic**	10.7%	43.8%	0 – 10	76.6	32.2	41.9 (23.4–77.3)

**Uganda**	6.5%	12.4%	20 – 73	75.3	20.6	25.2 (14.4–37.0)
**Togo**	6.7%	36.3%	0 – 100	82.2	19.1	22.1 (13.2–35.1)
**Cote d'Ivoire**	7.1%	45.8%	0 – 100	75.9	19.1	23.4 (14.2–39.1)

**Congo**	5.3%	54.4%	0 – 100	62.5	13.2	18.6 (9.8–29.3)
**Swaziland**	33.4%	23.9%	69 – 0	5.6	12.8	243.9 (122.1–402.3)
**Gabon**	7.9%	85.2%	0 – 96	38.5	12.5	30.7 (19.6–53.0)
**Nigeria**	3.9%	48.3%	1 – 99	70.8	12.0	16.7 (9.4–26.6)
**Guinea-Bissau**	3.8%	35.6%	0 – 100	78.9	11.4	13.9 (7.9–23.6)
**Cameroon**	5.4%	52.9%	24 – 74	53.3	11.3	20.1 (13.3–31.2)

**Chad**	3.5%	25.8%	14 – 86	76.3	11,2	15.1 (6.4–23.9)
**Gambia**	2.4%	26.1%	0 – 100	95.4	10.8	11.3 (5.8–19.0)
**Botswana**	24.1%	52.5%	13 – 0	4.9	9.3	197.6 (116.5–278.9)
**DRCongo**	3.2%	32.7%	10 – 85	73.7	9.2	12.2 (6.5–20.4)
**Liberia**	4%	47.9%	0 – 100	69.2	9.1	12.2 (7.3–21.5)

**Equat Guinea**	3.2%	50%	02–97	68.1	8.4	11.7 (7.1–18.0)
**Tanzania**	3.2%	37.5%	21 – 75	65.0	7.8	11.2 (5.8–19.4)
**Zimbabwe**	20.1%	35.9%	54 – 0	5.2	7.5	144.2 (75.6–221.0)
**Ghana**	2.3%	46.3%	02 – 98	76.1	7.5	9.2 (5.5–13.5)

**Namibia**	19.6%	33.5%	8 – 0	5.3	7.1	134.9 (74.4–247.1)
**South Africa**	18.8%	57.9%	15 – 0	5.0	6.8	137.0 (84.5–212.1)
**Burkina Faso**	2%	18.6%	0 – 100	94.9	6.5	6.8 (3.7–10.9)
**Sudan**	2.3%	40.8%	42 – 56	48.9	5.8	13.4 (4.2–28.3)
**Kenya**	6.1%	47.9%	57 – 21	22.8	5.7	25.6 (15.3–37.7)
**Angola**	3.7%	37.2%	53 – 46	38.9	5.7	14.7 (8.8–23.4)
**Mali**	1.7%	33.7%	10–90	72.7	5,0	6.5 (3.7–10.0)
**Guinea**	1.5%	36.5%	01–99	81.0	5.0	5.6 (3.3–8.3)
**Benin**	1.8%	46.1%	0 – 100	73.7	5.0	6.4 (3.6–10.4)

**Burundi**	3.3%	10.6%	64 – 21	29.9	4.1	13.5 (8.0–18.9)
**Niger**	1.1%	23.3%	11 – 89	79.4	2.8	3.5 (1.8–6.6)
**Sierra Leone**	1.6%	40.2%	0 – 100	78.9	2.7	3.6 (1.7–5.8)
**Senegal**	0.9%	51%	03–97	68.1	2.6	3.9 (2.1–6.4)
**Eritrea**	2.4%	20.8%	83 – 16	19.5	2.3	11.4 (5.8–19.8)
**Ethiopia**	2%	16.2%	50 – 14	25.7	2.0	8.2 (3.8–13.3)

**Rwanda**	3.1%	21.8%	60 – 7	14.3	2,0	13.8 (8.1–19.0)
**Mauritania**	0.7%	64.3%	59 – 41	25,4	1.3	5.3 (1.9–10.5)
**Madagascar**	0.5%	27%	36 – 60	58.3	1.1	2.0 (0.9–3.7)
**Somalia**	0.9%	35.9%	96 – 3	7.2	0.4	5.6 (2.5–9.2)

**Average Africa^$^**	6.1%		23 – 87	55.1	9.9	**18.0% (11.6–26.9)**
**Median Africa**	3.5%	36.5%	11–83	68.0	7.5	**14.1 (9.2–20.5)**

*Excluded from analyses were the following small countries: Seychelles, Reunion and Comoros, which are at negligible malaria risk and the islands of Sao Tome & Principe and Cap Verde, which are subject to stable transmission but have not been precisely characterized in the malaria risk model. Calculations used country-specific age distributions, based on HIV-1 prevalence estimates by UNAIDS/World Health Organization (WHO) for end of 2005[27], and assuming urban/rural ratios in HIV prevalence estimated by UNAIDS/WHO) for end of 2003 [27,28]

^† ^Urban and rural populations were based on countries' definitions[12], without standardization between countries.

^‡ ^Individual at risk is person living in a malaria prone area.

^$ ^Weighted according to countries' population size living in malaria endemic areas.

**Figure 2:**
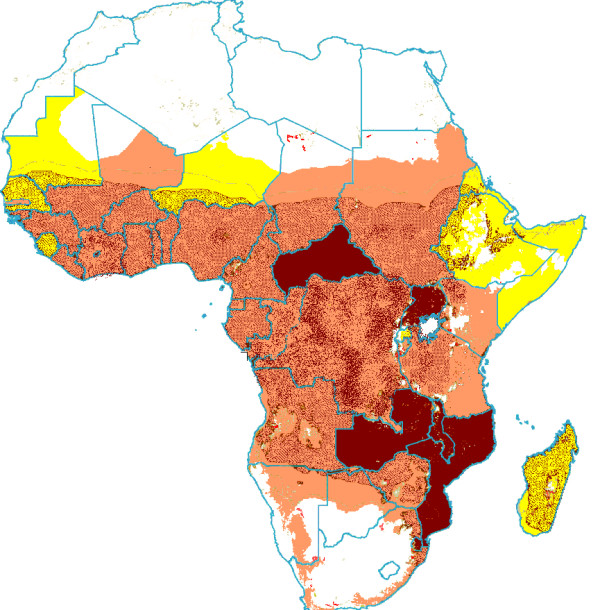
Additional parasite biomass due to HIV-1 in individuals living in malaria risk areas in absolute terms *. No Malaria Transmission . < 3 *10^9 ^parasites/individual at risk increase* . 3–20*10^9 ^parasites/individual at risk increase* . ≥ 20 *10^9 ^parasites/individual at risk increase* . *High Malaria Transmission areas shaded.

In absolute terms, HIV-1 infection increased the annual malaria parasite biomass from 4.08*10^19 ^(95%CI: 1.30–8.65*10^19^) to 4.86*10^19 ^(95%CI:1.48–10.63*10^19^). Within the countries, the median increase per person living in a malaria endemic area was 7.5*10^9^, ranging from 0.4*10^9 ^in Somalia to 54.3*10^9 ^in Mozambique (Table 3). The highest absolute increases were found in countries where both HIV prevalence and malaria endemicity were high (Table 3, Figure 2), i.e. Zambia, Malawi, Mozambique and the Central African Republic.

The sensitivity analysis (Table 4) shows that these estimates are sensitive to the magnitude of the impact of HIV-1 infection on the malaria incidence rates and associated parasite densities, with the assumptions for uncomplicated episodes being more influential than those for severe episodes. Excluding the effect of HIV-1 in children did not markedly reduce the estimate of biomass increase because in this age group the HIV-1 prevalence is low. For all alternative scenarios, the country ranking of absolute or relative increases in parasite biomass remained unchanged (not shown). Combining the uncertainty ranges on assumptions for all parameters presented in Table 1, the overall 95%CI for the estimated relative increase in parasite biomass would be 11.6–26.9% (best estimate 18.0%).

**Table 4:** Sensitivity analyses on the estimated relative increase in *Plasmodium falciparum *parasite biomass due to HIV-1, sub Saharan Africa*

			**Relative increase parasite** ** biomass**	
Best estimate (95% CI)*			**18.0%(11.6–26.9)**	

***Parameters***	***Lower-bound assumption***	***Upper-bound assumption***	***Lower-bound assumption***	***Upper-bound assumption***

HIV prevalence (UNAIDS country point estimate)	lower-bound	higher-bound	11.4%	23.8%
Relative risk of incidence of uncomplicated symptomatic malaria, in HIV-infected individuals with CD4<200/μl and CD4 200–499 compared to HIV-negatives	4/2	6/4	15.8%	19.4%
Relative parasite density during uncomplicated symptomatic malaria, in HIV-infected patients with CD4<200/μl and CD4 200–499/μL compared to HIV-uninfected patients	2/1	4/2	15.5%	19.9%
Relative risk of progression to severe malaria, in HIV-infected individuals with CD4<200/μl and CD4 200–499/μL compared to HIV-uninfected patients	8/3	24/12	17.2%	18.6%
Relative parasite density during severe malaria, in HIV-infected patients with CD4<500/μl compared to HIV-uninfected patients	0.5/0.5	2.0/2.0	16.9%	18.2%
Assume no HIV-1 impact on incidence of symptomatic malaria, severe malaria or parasite density in children under 15 years			17.6%	*n.a*.
Mean parasite density during uncomplicated symptomatic malaria in HIV-negative patients equal for all age groups and endemicities: 20000/μL			*n.a*.	17.2%

* For default values underlying this best estimate, see Table 2.

## Discussion

### Impact of HIV on parasite biomass

The HIV-1 epidemic may have increased malaria parasite biomass in sub-Saharan Africa by 17.4%. The highest absolute increases were found in countries with both high malaria and HIV burden (Zambia, Malawi, Mozambique and the Central African Republic). A recently published model [7] found that the largest epidemiological impact occurs when one baseline measure is very high while the other is very low and near its endemic threshold. When both prevalences were very high, the direct epidemiological impact of the interaction seemed to be minimal. It is indeed conceivable that, when both diseases reach an epidemic equilibrium, the epidemiological impact of the interaction will be minimal while the disease interaction should be maximal. Nevertheless, when both diseases are at epidemic equilibrium, there may be an indirect but important epidemiological impact such as the increased parasite biomass, higher biodiversity and higher probability of *de novo *resistance mutations emerging.

The model presented has several limitations. Pregnant women were not considered in the model as the parasite biomass in this high-risk group and its changes according to the HIV-1 infection could not be estimated, partly because of the sequestration of parasites in the placenta. Considering that pregnant women are particularly vulnerable to malaria infection and that such vulnerability is increased by HIV-1 infection, their exclusion may have led to an underestimation of the actual impact of HIV-1 infection at population level.

Moreover, the possible impact of HIV-1 infection on parasite density in asymptomatic individuals was not considered. This simplification may be justified because, due to the logarithmic distribution of malaria parasites in human populations, asymptomatic carriers, despite outnumbering the clinical cases, carry only a small proportion (probably less then 1/1000) of the world's potentially transmissible parasites [31,32]. In addition, asymptomatic infections most often are not treated, reducing the risk of selecting resistant parasites. In absence of drug pressure, mutant resistant parasites may be "less fit" and possibly less transmitted. However, once resistance has emerged and spread above a certain threshold, asymptomatic infections could play a substantial role for its further spread, especially in high endemic areas, and this effect could be enhanced by a higher prevalence of asymptomatic infection in HIV co-infected individuals. The spread of these resistant strains should be calculated by a stochastic model where asymptomatic carriers most probably play an important role.

An additional limitation of the model presented is the assumption that the effect of the HIV-1 infection on parasite density is constant throughout the clinical episode. This may not be true as the actual parasite density may vary throughout the clinical episode.

### Impact of HIV on the emergence of antimalarial drug resistance?

Several factors determine the probability of selecting the *de novo *mutations linked to antimalarial drug resistance [33]. Amongst them, the frequency at which the resistance mutation occurs and the number of parasites in the human host exposed to the drug are considered as independent risk factors. If the probability of *de novo *resistance mutations is distributed evenly among all parasites then the biomass increment due to HIV-1 directly increases the rate at which these mutations occur.

**Figure 3:**
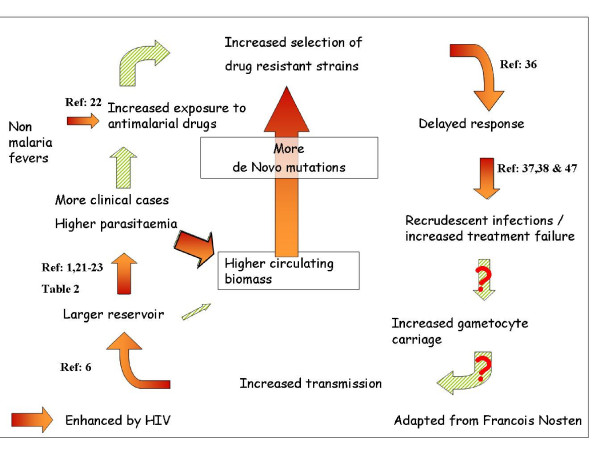
Effects of HIV on the emergence and consequent spread of antimalarial drug resistance in human populations.

Mathematical models of malaria epidemiology and drug resistance represent important tools in guiding malaria control strategies. They consistently predict that once drug resistance arises, it spreads rapidly, with the level of host immunity (both aspecific and specific) considered as an additional important independent factor. Although the dynamics underlying the spreads of antimalarial resistance may be much more complex than previously realized [34], the cycle of the emergence and subsequent spread of antimalarial drug resistance [35], and the influence of HIV-1-related immune-suppression can be schematically represented (Figure 3). Drug resistance starts with one or several genetic mutations in parasites which, if exposed to the drug, are selected and may spread. HIV-1 infection contributes to the emergence and spread of drug resistance by increasing drug exposure and drug pressure. HIV-1 infection increases the probability of a malaria infection progressing to symptomatic illness and to a higher parasite densities, increasing the probabilities of treatment and contact between the parasites and the drug [1,21-23]. In addition, immune suppressed HIV-infected adults suffer frequently of non-malaria-attributable acute fevers that may be misdiagnosed as malaria and treated as such [22]. Once resistance has emerged and spread above a certain threshold, malaria treatment of non-malaria fever in HIV immune-suppressed individuals might contribute to the further spread of drug resistance by increasing the drug pressure on asymptomatic malaria infections. After treatment, a delayed cure rate [36] and a higher rate of recrudescence [37,38], a phenomenon already described in HIV-1 infected individuals, accelerate the spread of resistant parasites and increase the parasite biomass in symptomatic patients and asymptomatic carriers alike.

Besides contributing to the emergence and spread of antimalarial drug resistance, HIV-1 infection may influence and modify its expected geographical pattern. It has been postulated that antimalarial drug resistance first emerges and spreads in low-endemic areas, where the population acquired immunity is so low that most infections are symptomatic and thus treated, exposing a large numbers of parasites to antimalarial drugs [39]. In highly endemic areas, less malaria infections become symptomatic and are treated (especially in adults). In sub-Saharan Africa, when considering the geographical distribution of both the HIV-1 infection and the spread of drug resistance (to chloroquine and sulphadoxine-pyrimethamine) [33,40], the latter having been faster in eastern-southern (high HIV-1 prevalence) than in western Africa (relatively low HIV-1 prevalence) [41], the question on the role of HIV-1 pandemic as an additional contributing factor could be raised. It is impossible to provide a definitive answer though the results of the model presented in this paper are compatible with an influence of HIV-1 infection on the epidemiology of antimalarial drug resistance.

Malaria parasite biomass is influenced through various malaria control interventions. Easy access, prompt diagnosis and efficacious treatment with combination therapy are essential to halt or delay the emergence and spread of antimalarial drug resistance [9]. The artemisinin derivatives, commercialized in the last decade, are the most potent antimalarial drugs with parasite reduction levels of 10,000 per cycle. Furthermore, if used in combination, the artemisinin component is protected by the partner drug and the parasite reduction rate is positively affected. Unfortunately, it is still unclear how treatment combinations for both malaria and HIV-1 interact. More data on the biological effects of therapy combinations would be of great utility to complement recent '*in vitro' *evidence of a synergistic effect between protease inhibitors and antimalarials [32]. Preventive interventions are equally important as they diminish the parasite load, transmission and drug pressure. Indeed, the incidence of symptomatic malaria in HIV-infected adults can decrease by 95% by giving cotrimoxazole prophylaxis and antiretroviral treatment combined with insecticide-treated bed nets [42]. Furthermore, next to individual protection, preventive activities as indoor residual spraying, use of bed nets and cotrimoxazole prophylaxis reduce the parasite biomass at community level. For indoor residual spraying this is quite obvious as it is a community intervention but it has been shown that cotrimoxazole prophylaxis taken by HIV-infected people was associated with decreased morbidity and mortality among family members [43]. Recent modeling has demonstrated that insecticide-treated nets protection has a substantial impact on the circulating malaria parasite biomass and has community-wide benefits that are often underestimated [44]. Therefore, during the ongoing scale-up of HIV/AIDS and malaria control measures, HIV-1 infected individuals should be considered a priority group for the distribution of insecticide-treated nets, co-trimoxazole prevention and artemisinin-based combination therapy.

## Conclusion

Earlier studies on the impact of HIV infection on malaria morbidity and mortality focused on the direct effects in HIV-infected people. They concluded that the population-level effect was limited because of different geographical distribution and contrasting age patterns of the two diseases. However, the model presented suggests that the overall impact of HIV-1 infection may be greater than previously estimated. If HIV-1 is a major contributing factor for the emergence of antimalarial drug resistance, this will have a major impact over the whole population by increasing malaria morbidity and mortality. A second stochastic model dealing with the contribution of HIV-1 infection on the spread of antimalarial drug resistance once resistance has emerged is currently under development.

Preventive interventions, easy access, prompt diagnosis and efficacious treatment with combination therapy are essential to diminish the parasite biomass, transmission and drug pressure and halt or delay the emergence and spread of antimalarial drug resistance [9]. This is even more important for HIV-1 infected malaria patients. Targeting this vulnerable group will greatly contribute to malaria control and will benefit the whole population in endemic countries.

## Authors' contributions

JPVg developed the model, did the literature research, did the analyses and wrote the paper. JM worked out the model in R^®^. RC reviewed the paper. EK contributed to analyses and writing of the paper. UDA contributed to the data interpretation and the writing of the paper. All authors read and approved the final manuscript.

## Conflict of interest stated

The authors declare that they have no competing interests.
